# A new strategy for the passive skin delivery of nanoparticulate, high molecular weight hyaluronic acid prepared by a polyion complex method

**DOI:** 10.1038/s41598-018-20805-3

**Published:** 2018-02-05

**Authors:** Yoshihiro Tokudome, Tatsuya Komi, Ami Omata, Marie Sekita

**Affiliations:** Laboratory of Dermatological Physiology, Department of Pharmaceutical Sciences, Faculty of Pharmacy and Pharmaceutical Sciences, Josai University, 1-1, Keyakidai, Sakado, Saitama 350-0295 Japan

## Abstract

Restoring hyaluronic acid (HA) content is important for maintaining the function of photo-aged skin. This study aimed to evaluate the passive delivery into skin of HA nanoparticles formed by the polyion complex method. Nanoparticles were prepared by mixing and stirring anionic HA with a cationic polymer, protamine, at the charge ratio 55:45. The permeation of fluorescently-labelled HA nanoparticles (HANP) or free HA through hairless mouse skin was characterized *in vitro*. HANP or free HA was applied to ultraviolet (UV)-irradiated mice *in vivo*, and their transepidermal water loss (TEWL) was measured after 4 days. HA that had been delivered into skin was separated and characterized by molecular sieve chromatography. HANP were able to deliver HA into the dermis both *in vitro* and *in vivo*, whereas free HA penetrated no further than the stratum corneum. Following HANP application, HA within the skin was present in the form of free HA rather than nanoparticles. When applied *in vivo*, HANP significantly reduced the TEWL caused by UV irradiation. Thus, although free HA does not penetrate into the skin by passive diffusion, HA can be effectively delivered by nanoparticles. HA is then released from the nanoparticles and can contribute to barrier recovery following UV irradiation.

## Introduction

The barrier function of skin is principally attributed to the stratum corneum. Only small molecules, usually less than 500 Da, and lipophilic compounds can penetrate the skin barrier^1^. Hyaluronic acid (HA), which is composed of repeated β-1,4-glucuronic acid–β-1,3-N-acetylglucosamine disaccharide units, is a non-sulfated glycosaminoglycan with a molecular weight of over 1,000 kDa^2^. HA is abundant in the extracellular matrix of the skin. HA is found in extracellular spaces, but also interacts with cells in many biological processes such as tissue homeostasis, cell proliferation^3^, cell migration^4^, cell differentiation^5^, angiogenesis^6^, tumor biology^7^, and anti-apoptosis^8^. The amount of HA in the skin is equivalent to 50% or more of the total amount of HA in the body^2^. It is well known that the HA content is decreased in skin that has been photo-aged by ultra violet (UV) irradiation^9,10^. Decreased HA has been suggested to lower the elasticity of skin, and promote the formation of wrinkles. It is therefore important to restore HA decreased by UV irradiation to normal levels, to maintain skin function. Many researchers have examined delivering HA into the skin. However, HA does not permeate through the skin because it is a water-soluble macromolecule. Therefore, when HA is administered to the skin, it remains on the skin surface and functions as moisturizing of the skin surface. However, many researchers want to deliver HA to deeper skin. HA injection has been widely used for delivery into the skin. It is medical practice. This method is not good as it is noninvasive. It has been reported the HA can be delivered into the skin by micro-needles and iontophoresis. Recently, the solid-in-oil (S/O) technique has been used to deliver high molecular weight compounds into the skin^11^. S/O nanodispersions are nanosized drug carriers designed to overcome the skin barrier. Kitaoka *et al*. discuss the rationale for preparing efficient and stable S/O nanodispersions, as well as examples of their application to deliver cosmetic and pharmaceutical materials, including vaccines. Drug administration using a patch is user-friendly, and may improve patient compliance. This technique is a potent transcutaneous immunization method not requiring needles. However, there are few reports that it is able to deliver materials to the deeper skin, where HA is found. Moreover, there are several reports on hyaluronic acid nanoparticles. However, in these reports, hyaluronic acid is not singly used, but by derivatizing hyaluronic acid particles are created and applied to cancer treatment^12,13^. In addition, methods of coating the surface of nanoparticles with hyaluronic acid by the polyion complex method, etc. have also been studied, but there is no report that hyaluronic acid is considered as an active ingredient. Also, there is no report using hyaluronic acid nanoparticles as a transdermal absorption enhancement.

Alternative microsphere structures prepared from plasmid DNA and chitosan with chondroitin sulfate have been reported^14^. Similarly, Ito *et al*. reported that nanoparticles can be formed by mixing plasmid DNA, polyethyleneimine and HA^15^.

We aimed to deliver hyaluronic acid into the skin beyond the limit of skin penetration. We have therefore prepared nanoparticles by mixing negatively charged HA with positively charged protamine (PRT). The penetration into the skin of these HA nanoparticles (HANP) was compared *in vivo* and *in vitro* with non-nanoparticulate HA. The ability of the HANP to reduce UV-induced skin damage in mice was also evaluated.

## Materials and Methods

### Materials

Sodium HA (FCH-120, MW 1,200 kDa, Purity: 99.9%) was obtained from Kikkoman Biochemifa Company (Tokyo, Japan). PRT was obtained from WAKO Pure Chemical (Osaka, Japan). 5-Fluoresceinamine, acetaldehyde and cyclohexyl isoniazide was purchased from Sigma (St. Louis, MO, USA). Pemulen TR-2^®^ was obtained from Lubrizol (Lakeland Boulevard Wickliffe, OH, USA). Diisopropyl adipate and squalane were kindly gifted by Nikko Chemicals Co. Ltd. (Tokyo, Japan). Other reagents were of analytical grade and used without further purification.

### Preparation of HANP

Aqueous solutions of anionic HA (average molecular weight: 1,200 kDa) and cationic PRT were mixed at each concentration for 15 min at room temperature. The samples were then passed through a 0.22 µm polyvinylidene difluoride filter (Milex-GV, Merck Millipore Ltd, Darmstadt, Germany).

### Measurement of zeta potential, particle diameter and polydispersity index (PDI) of HANP

The zeta potential, particle diameter and PDI for HANP were determined using a Zetasizer Nano-ZS instrument (ZEN3600, Malvern Instruments, Malvern, Worcestershire, UK).

### Observation of HANP by transmission electron microscopy

The structure of HANP was analyzed using a transmission electron microscope, with negative staining (JEM 1010, Jeol, Tokyo, Japan). Scale bars indicate 100 µm.

### Particle diameter and PDI change due to coexisting ions

Various concentrations of PBS and HANP were mixed and determined after 24 h by Zetasizer Nano-ZS.

### Preparation of fluorescence labeled HA

Fluorescence labeled HA (FL-HA) was prepared according to the method of Belder and Wik^16^. Briefly, FL-HA was prepared by the condensation reaction of four reagents (aldehyde, amine, isocyanide and carboxylic acid). The HA was dissolved in 40 mL of purified water (1.25 mg/mL), and 20 mL of dimethylsulfoxide (DMSO) was added. Next, 0.5 mL of 5-fluoresceinamine (50 mg/mL) in DMSO, 25 µL of cyclohexyl isocyanide and 25 µL of acetaldehyde were added to the HA solution. The mixture was stirred using a stirrer for 5 h. Saturated sodium chloride solution and ice-cold ethanol were then added to the mixture. It was then centrifuged (4,000 × g, 10 min) and the precipitate was collected. The precipitate was dissolved in purified water, placed in cellulose dialysis tubing (molecular weight cut off, 14 kDa; pore size, 50 Å) and dialyzed for 24 h to remove unreacted 5-fluoresceinamine. The resulting FL-HA solution was freeze-dried (FZ-6, Labconco, Kansas City, MO, USA) to obtain FL-HA powder.

### Preparation of fluorescein-labeled HANP emulsions

Solutions of anionic FL-HA (average molecular weight: 1,200 kDa) in distilled water and aqueous, cationic, unlabeled PRT were mixed for 15 min at room temperature. The resulting fluorescein-labeled HANP (FL-HANP) suspensions were freeze-dried and re-suspended in distilled water, as the required FL-HANP concentration was five times that of original preparation. Next, 1,3-butanediol and Pemulen TR-2^®^ (emulsifier) were added and mixed at 5,000 rpm at 60–70 °C (Labolution^®^, PRIMIX Corporation, Awaji, Hyogo, Japan). Finally, potassium hydroxide and isopropyl diadipate or squalane (oil phase) were added, and mixed at 10,000 rpm.

### Animals

Male hairless mice (Hos:HR-1 strain, 7 weeks old, 20–25 g) were purchased from Hoshino Laboratory Animals, Inc. (Bando, Ibaraki, Japan). Mice were housed under a 12 h light and dark cycle in a temperature-controlled room (25 °C). They had free access to food and water. All animal procedures were approved by the Ethics Committee of Josai University (Sakado, Saitama, Japan) in accordance with the National Institute of Health (Tokyo, Japan).

### Preparation of skin membranes

Penetration studies were conducted with excised full-thickness hairless mouse skin (7 weeks old). To obtain stratum corneum-stripped skin, adhesive tape was applied to hairless mouse skin with uniform pressure and then removed. This procedure was repeated approximately 20 times, until the SC was entirely removed. Subcutaneous fat was carefully eliminated from all skin specimens.

### *In vitro* penetration study

Excised specimens of full-thickness or stripped skin were mounted in a modified Franz-type diffusion cell having an effective diffusion area of 1.77 cm^2^. Test solutions (1.0 mL) were placed on the stratum corneum side of the skin. The receiver solution was 5.0 mL of phosphate buffered saline (PBS, pH 7.4), which was maintained at 32 °C. After 24 h (stripped skin) or 48 h (full-thickness skin), frozen sections were prepared from the skin, and fluorescence derived from FL-HA and FL-HANP was observed using a confocal laser scanning microscope (FV1000-D, Olympus Corporation, Tokyo, Japan).

### Determination of FL-HA in skin

After the penetration experiment, each skin sample was cut into several pieces and then homogenized in 10 mL methanol. The quantity of FL-HA was assayed by fluorescence high performance liquid chromatography (HPLC), as described below.

### HPLC conditions

Determination of FL-HA was performed using a fluorescence HPLC system. The mobile phase was 10 mM ammonium acetate containing methanol (8:2). HPLC separation was performed using a Prominence system (Shimadzu, Kyoto, Japan) with a YMC-Pack Diol-300 column (300 × 8.0 mm, S-5 mm, 30 nm) from YMC CO., LTD. (Kyoto, Japan). The flow rate was 1.0 mL/min. The fluorescence detector (RF-A_XL_, Shimadzu) was set at excitation: 494 nm, emission: 521 nm.

### Change in transepidermal water loss of UV-B irradiated mice after application of HA or HANP

Mice were randomly assigned to four groups: normal, control, HA and HANP (four mice per group), each receiving a different topical treatment (Table 1). Each treatment was applied every day (200 µL). Animals in the groups receiving UV-B irradiation were exposed to 60 mJ/cm^2^ applied with a Philips Broadband TL 20w/12RS lamp (Philips, BC, Amsterdam, Netherlands). Transepidermal water loss (TEWL) was measured in triplicate for each mouse, and the mean values were obtained. A VAPO SCAN AS-VT100RS instrument (Asahi Biomed, Yokohama, Japan) was used to measure cutaneous water evaporation. The procedure was performed five times, on the last day of the experiment.

**Table 1:** The *in vivo* experimental groups.

Group	Topical treatment	Applied volume (mL)	UV-B irradiation (mJ/cm^2^)
Normal	None	None	0
Control	Emulsified preparation	0.2	60
HA	HA (emulsion)	0.2	60
HANP	HANP (emulsion)	0.2	60

### Statistical analysis

Analysis was performed using Statistical Analysis SAS statistical software version 9.2 (SAS Institute, Cary, NC). Indicated *p*-values were derived from the Student’s t-test or Tukey’s post-hoc multiple comparison test.

## Results

### Preparation and characterization of HANP

**Figure 1:**
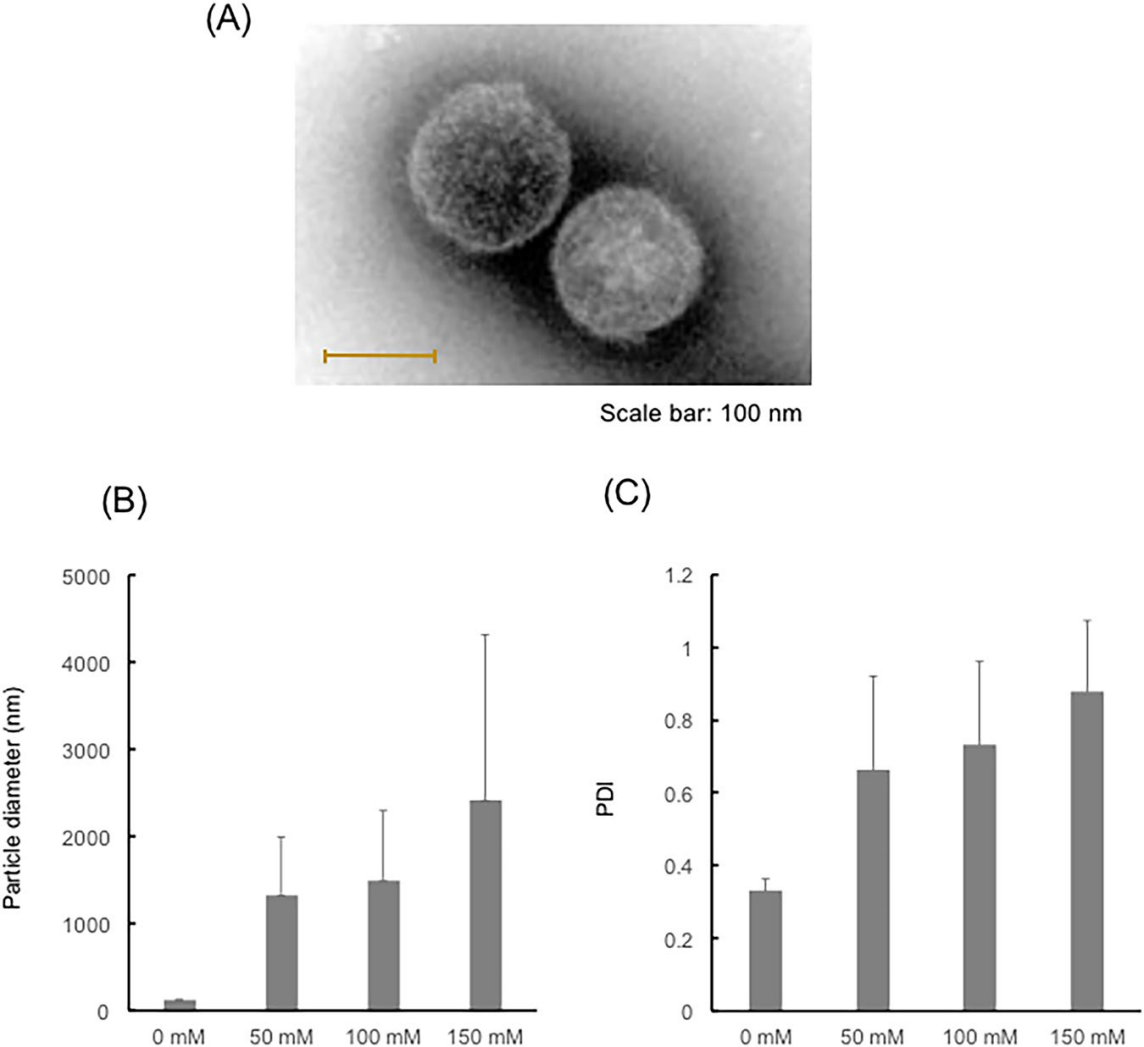
Characterization of hyaluronic acid nanoparticles. Transmission electron microphotograph image of hyaluronic acid nanoparticles (**A**). The structure of HANP was analyzed using a transmission electron microscope, with negative staining. HANP with a charge ratio of 55:45 are shown. Scale bars indicate 100 nm. Particle diameter (**B**) and PDI (**C**) change due to coexisting ions. Various concentrations of PBS and HANP were mixed and determined after 24 h by Zetasizer Nano-ZS.

HANP were prepared by setting the final, combined concentration of HA and PRT to be 0.1 mM, while varying the ratio of HA to PRT. The characterization of the resulting nanoparticles is summarized in Table 2. The polydispersity index (PDI) was minimized when HA:PRT was 55:45. Figure 1A shows a transmission electron microphotograph of nanoparticles with HA:PRT = 55:45, which suggests that the HANP had a core–shell structure. FL-HANP were prepared only at an FL-HA:PRT ratio of 55:45, and showed the same physical properties as unlabeled HANP with the same charge ratio (data not shown). Various concentrations of PBS and HANP were mixed and determined after 24 h by Zetasizer Nano-ZS. Particle diameter (Fig. 1B) and PDI (Fig. 1C) change due to coexisting ions.

**Table 2:** Characterization of FL-HANP.

Composition (HA:PRT%)	Particle Diameter (nm)	Zeta potential (mV)	Polydispersity index
10:90	109.7	20.4	0.371
20:80	108.4	20.1	0.387
30:70	112.5	18.2	0.347
40:60	102.2	18.1	0.274
45:55	101.8	18.1	0.240
50:50	113.8	15.4	0.186
55:45	137.0	−30.2	0.127
60:40	118.0	−36.4	0.136
70:30	117.3	−44.7	0.241
80:20	121.7	−52.7	0.440
90:10	151.7	−51.2	0.568

### *In vitro* skin penetration of aqueous FL-HA and FL-HANP

**Figure 2:**
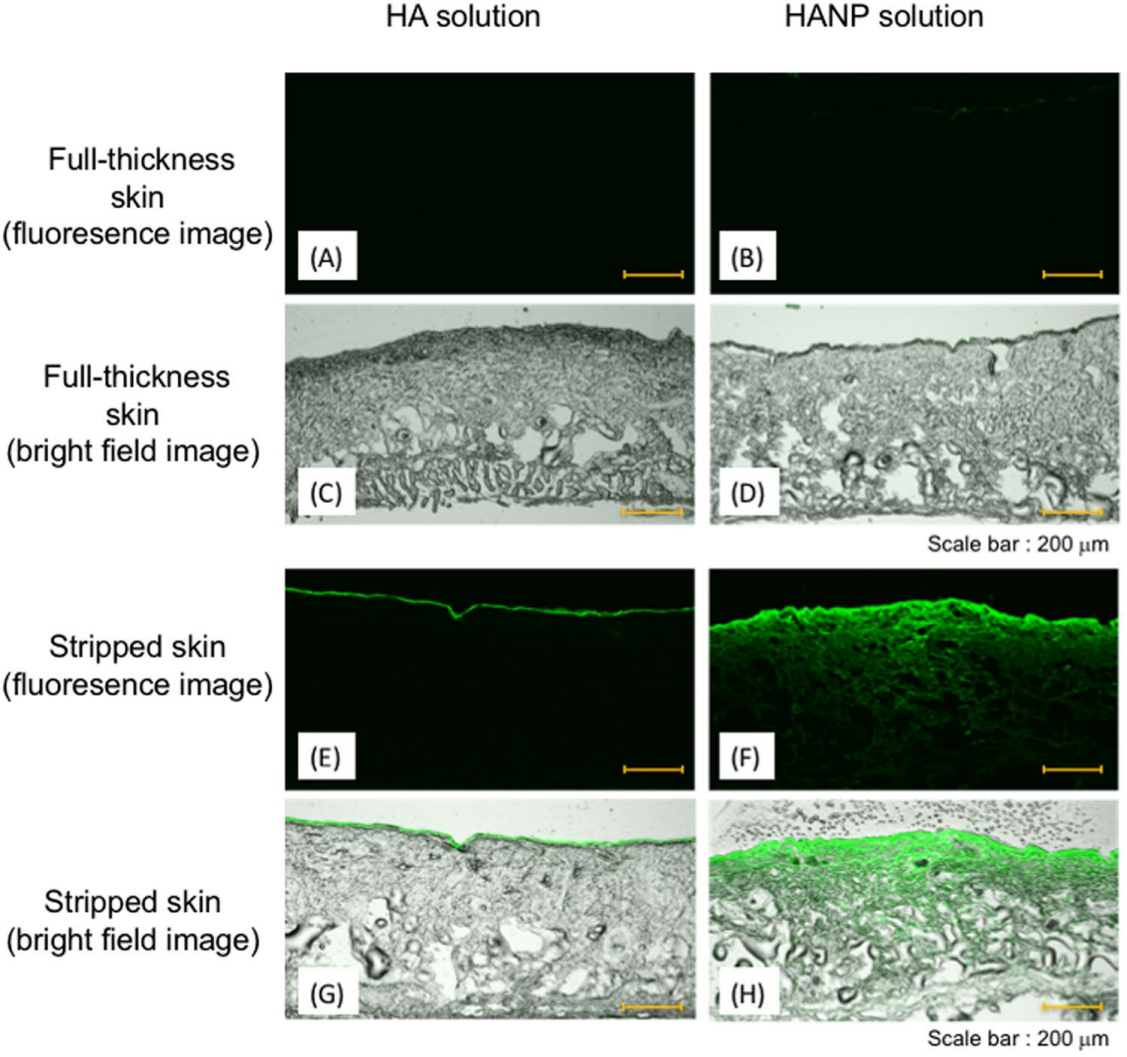
*In vitro* skin penetration of aqueous FL-HANP or FL-HA. Male HR-1 skin was mounted in a modified Franz-type diffusion cell. Twenty-four hours (stripped skin, **E**,**F**,**G** and **H**) or 48 h (full-thickness skin, **A**,**B**,**C** and **D**) after adding the donor solution, a frozen skin section was prepared, and fluorescence derived from FL-HA (**A**,**C**,**E** and **G**) or FL-HANP (**B**,**D**,**F** and **H**) was observed by confocal laser scanning microscopy. Bright-field images are shown in (**A**,**B**,**E** and **F**), and fluorescence images in (**C**,**D**,**G** and **H**). Scale bars indicate 200 µm.

The effects of the stratum corneum on skin penetration by aqueous FL-HA solutions or FL-HANP suspensions were investigated using a diffusion cell. Figure 2 shows the penetration of FL-HA or FL-HANP into full-thickness skin or stripped skin. Aqueous FL-HA and FL-HANP did not penetrate full-thickness skin. In stripped skin, the aqueous FL-HA solution remained near the outer surface. In contrast, the FL-HANP suspension penetrated deeper into the stripped skin.

### *In vitro* skin penetration of FL-HANP emulsions

**Figure 3:**
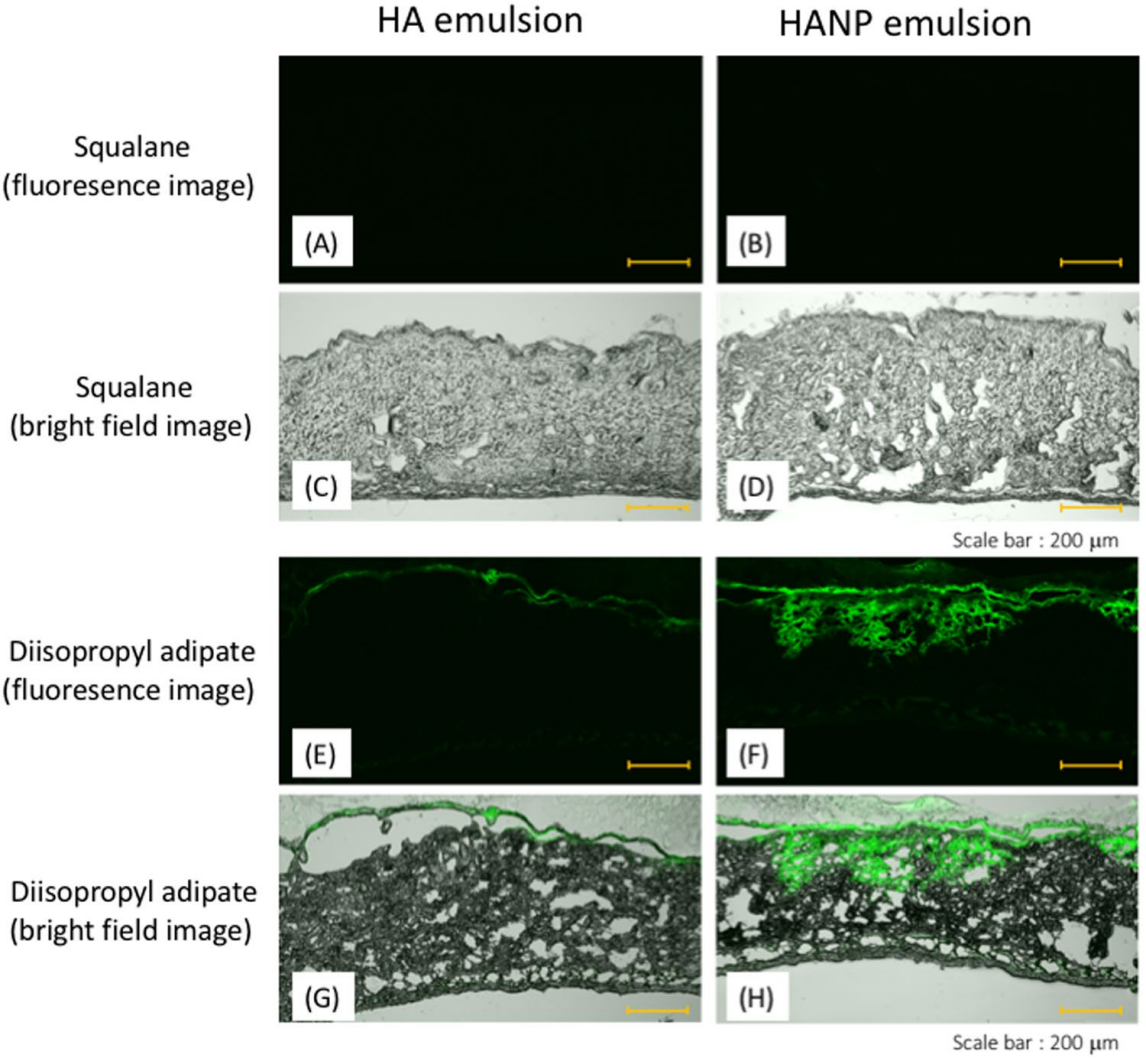
*In vitro* skin penetration of FL-HA or FL-HANP emulsions. Full-thickness male HR-1 skin was mounted in a modified Franz-type diffusion cell. Donor formulations were prepared using emulsions containing diisopropyl adipate (**E**,**F**,**G** and **H**) or squalane (**A**,**B**,**C** and **D**). Forty-eight hours after adding the donor solution, a frozen skin section was prepared, and fluorescence derived from FL-HA (**A**,**C**,**E** and **G**) or FL-HANP (**B**,**D**,**F** and **H**) was observed by confocal laser scanning microscopy. Bright-field images are shown in (**A**,**B**,**E** and **F**), and fluorescence images in (**C**,**D**,**G** and **H**). Scale bars indicate 200 µm.

The penetration through full-thickness mouse skin of non-nanoparticulate FL-HA or FL-HANP emulsified in diisopropyl adipate or squalane (oil phase) and Pemulen TR-2^®^ was evaluated. For the emulsions with squalane and Pemulen TR-2^®^, fluorescence of FL-HA origin was not observed in the skin at 48 h after application of either non-nanoparticulate FL-HA or FL-HANP. On the other hand, emulsions containing diisopropyl adipate did penetrate into the skin, with fluorescence derived from FL-HA observed in the stratum corneum for non-nanoparticulate FL-HA and also deeper in the skin for FL-HANP (Fig. 3).

### Analysis of aqueous FL-HA and FL-HANP by fluorescence HPLC

**Figure 4:**
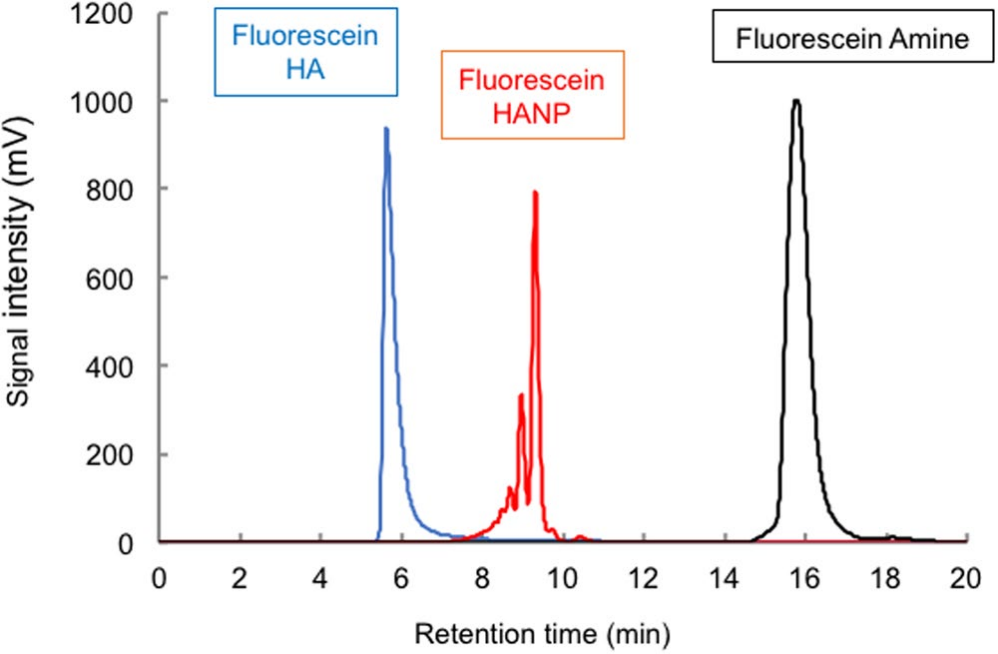
Fluorescence HPLC analysis of aqueous FL-HA or FL-HANP. Determination of FL-HA was performed using a YMC-Pack Diol-300 gel filtration chromatography column and a fluorescence HPLC system. An HPLC chart of FL-HA and FL-HANP elution is shown.

Figure 4 shows an HPLC chromatogram of non-nanoparticulate FL-HA and FL-HANP. Their retention times were different. A positive correlation was found between the concentration and the area under the chromatogram peak for both non-nanoparticulate FL-HA and FL-HANP (data not shown).

### FL-HA content in skin after application of FL-HANP *in vitro*

**Figure 5:**
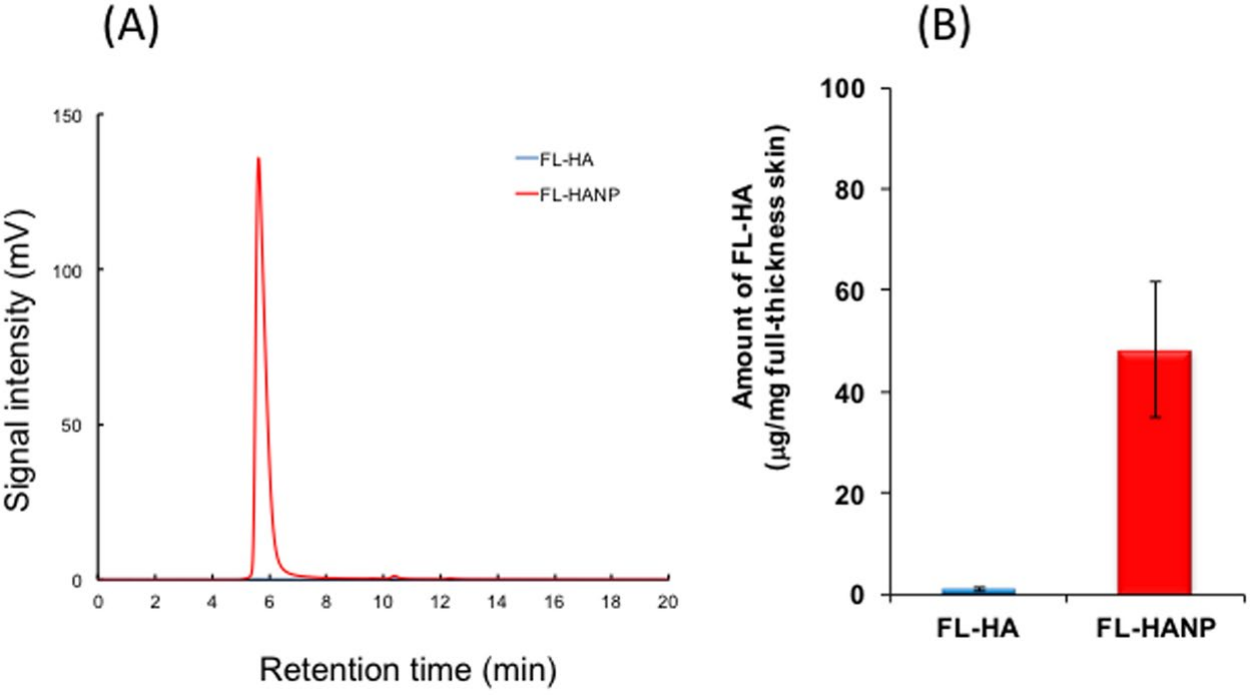
FL-HA content in skin after application of FL-HANP *in vitro*. The skin was attached to a Franz-type diffusion cell and an FL-HA or FL-HANP emulsion was applied. Forty-eight hours after the emulsion was applied, each skin sample was collected, cut into several pieces and then homogenized. The FL-HA content in skin was assayed at excitation: 494 nm, emission: 521 nm. An HPLC chart of FL-HA in skin after application of FL-HA or FL-HANP emulsions is shown in (**A**). Quantitation of FL-HA in skin is shown in (**B**). Values are expressed as the means ± standard deviations (*n* = 3). *p < 0.05, Student’s *t*-test.

By using the analysis method above, FL-HA in the skin was measured at 48 h after the application of non-nanoparticulate FL-HA or FL-HANP emulsions (Fig. 5A). FL-HA content was 0.1 or 45 µg/mg full-thickness skin in the non-particulate FL-HA or FL-HANP application group, respectively. Hence, FL-HA content was significantly increased following the application of FL-HANP rather than non-nanoparticulate FL-HA (*p* < 0.05). In contrast to FL-HANP before application to skin (Fig. 4A), the retention time of FL-HANP that had been recovered from the skin was the same as that of non-nanoparticulate FL-HA (Fig. 5B).

### Measurement of TEWL after application of HA or HANP emulsion *in vivo*

**Figure 6:**
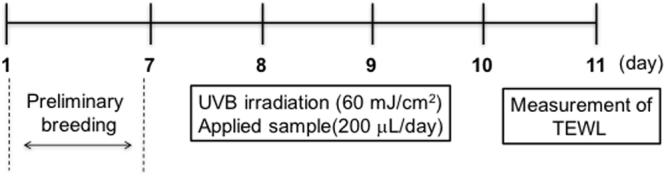
Schedule of an experiment evaluating the effects of HA and HANP on TEWL. Preliminary breeding of mice required 7 days. The hairless mice were then irradiated with UV-B (60 mJ/cm^2^) continuously for 4 days. Each HA or control preparation was applied every day (200 µL). TEWL of mice was measured after 4 days.

**Figure 7:**
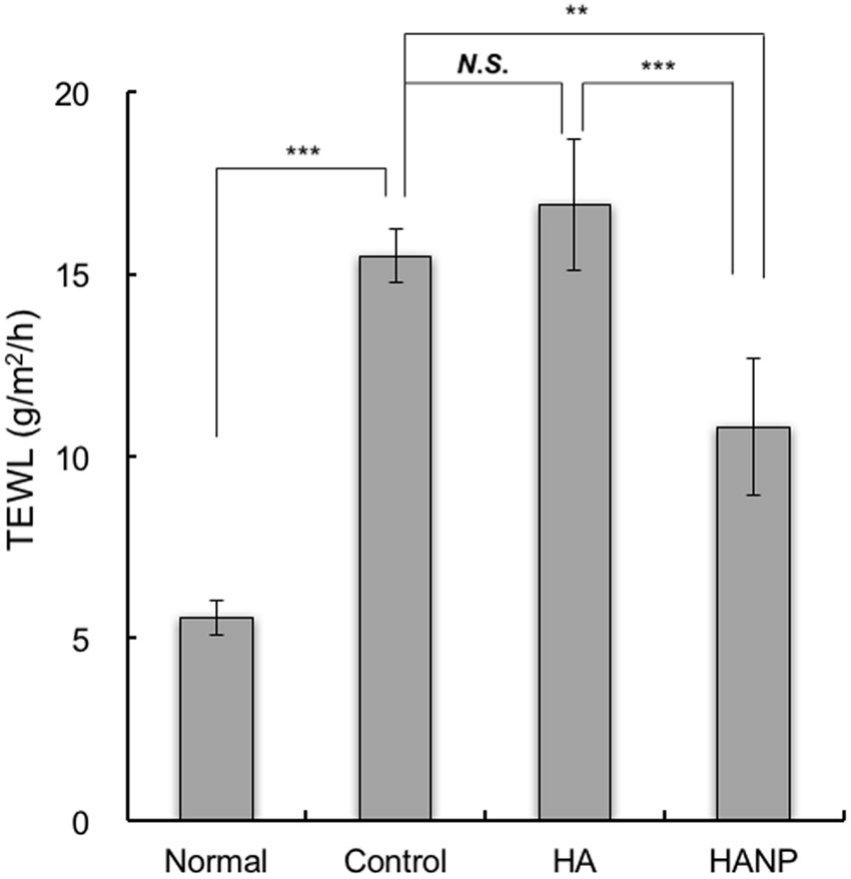
Changes in TEWL after application of HA or HANP *in vivo*. TEWL was measured after 4 days continuous UV-B exposure. Values are expressed as the means ± standard deviations (*n* = 4). **p < 0.01, ***p < 0.001, Tukey’s multiple comparison test.

Hairless mice were irradiated with UV-B (60 mJ/cm^2^) continuously for 4 days. TEWL was then measured after 4 days’ irradiation. The experimental groups are shown in Table 2 and the test schedule is shown in Fig. 6. The results are shown in Fig. 7. TEWL in the normal group (not irradiated) did not vary significantly throughout the study. However, TEWL increased to approximately 15 g/m^2^/h in the control and non-nanoparticulate HA groups (UV-B irradiated). In contrast, TEWL in the HANP group (also UV-B irradiated) was significantly lower than in the control and non-particulate HA groups.

## Discussion

The stratum corneum is the outermost layer of the skin and constitutes the greatest barrier. It consists of keratinocytes and intercellular lipids. Many compounds penetrate the skin via the intercellular lipids. It is assumed that the spaces between intercellular lipids of the stratum corneum are narrow. Recent studies have reported that liposomes with negative charges show improved skin penetration because of their small particle size^17^. Therefore, we hypothesized that small, negatively charged and stable particles could be delivered into the skin.

In this study, HA was granulated by the polyion complex method with the purpose of improving skin penetration. Firstly, the ratio of anionic FL-HA to cationic PRT that was used to form particles was varied. Particle formation was confirmed over a wide range of ratios. In addition, the surface charge of the nanoparticles varied depending on the ratio of FL-HA to PRT. When the ratio was 55:45, the PDI was lowest, and particles with a surface potential of approximately −30 mV could be prepared at a size of approximately 100 nm.

Skin penetration was evaluated using aqueous suspensions of these HANP. Fluorescence derived from FL-HANP was not observed following application to full-thickness skin. In general, it is known that the skin penetration of a compound having a molecular weight exceeding 500 kDa is extremely low^1^, and it has been found that aqueous HA and HA particles do not penetrate into the skin. Therefore, an *in vitro* skin penetration test using stripped skin was performed. Fluorescence derived from FL-HA was not found following the application of aqueous non-nanoparticulate FL-HA, but was observed when FL-HANP were applied. Although the details are unknown, it is inferred that partitioning into the living layers of epidermis or diffusion into the skin was improved by HANP formation.

Next, the penetration of HA or HANP emulsions into full-thickness skin was evaluated. No fluorescence derived from FL-HA was observed following the application of non-nanoparticulate FL-HA emulsions. However, when FL-HANP emulsions were applied, fluorescence derived from FL-HA was observed in the deeper skin only when diisopropyl adipate was used to prepare the emulsion. Emulsified formulations can contain surfactants, oils, polyhydric alcohols, water and other compounds. There are reports that surfactants^18^, oils^19^ and polyhydric alcohols^20^ promote the skin penetration of low molecular weight compounds. However, it is not clear that the same applies to high molecular weight polymers. Despite the failure of non-nanoparticulate HA emulsions to deliver HA into the skin, the reason why a granulated HA emulsion could deliver HA is unknown. It is also interesting that the skin penetration of HANP varied when different oils were used to prepare the emulsions. Detailed investigation of the percutaneous absorption route would be desirable in the future.

More interestingly, when FL-HA was extracted from the skin and quantitated by HPLC using a size exclusion column, the retention time of the fluorescent compound extracted from the skin was not the same as that of the FL-HANP before application to skin. The retention time was the same as that of non-nanoparticulate FL-HA. It has been confirmed that HA nanoparticles are not destroyed by organic solvents. However, these results suggest that the HA was present as nanoparticles when applied to the skin, but as a non-nanoparticulate molecules after penetration into the skin. In our result, particle diameter and PDI greatly change due to coexisting ions (Fig. 1(B) and (C)). This indicates that the particles are unstable. Nanoparticles in the skin may be destroyed by ions in the body and exist as non-particle HA. The physiological actions of HA include suppression of angiogenesis^21,22^, anti-inflammatory activity^23,24^, and roles in skin development, proliferation, differentiation^25^ and regeneration^26^. It is extremely interesting to anticipate that HA could be applied to skin as nanoparticles, but could then act within the skin as free HA molecules.

Next, the effect of HANP on improving skin barrier function was evaluated. The application of an HANP emulsion to continuously UV-B irradiated mice suppressed the increase in TEWL. These results suggest that HANP improve the skin barrier function that is reduced by UV-B exposure. It has been reported that HA promotes differentiation^25^, and as a result, the content of ceramide and similar molecules in the stratum corneum is increased and TEWL is thereby supposed to be decreased.

## Conclusion

We have shown that polymeric HA can be formulated into nanoparticles using the polyion complex method. Although free HA cannot be delivered into the skin, we found the application of an emulsified preparation of HANP led to the passive diffusion of HA into the skin. Furthermore, the increase in TEWL due to UV-B irradiation could be suppressed by applying these HANP. Even more interestingly, our results suggest that HA is released from the nanoparticles to form free molecules following delivery into the skin.

Hence, by using HANP, HA can be delivered into the skin at high concentration. The finding that HA can be delivered into the skin without using injection or physical skin penetration indicates that this method could prove highly useful as a substitute for HA injection. The skin penetration route involved and the delivery of polymers other than HA would be worthy of future examination.
